# Northern Norway Sporophytes of *Saccharina latissima* Display Distinct Gene Expression Profiles in Response to Temperature and Photoperiod

**DOI:** 10.1002/ece3.71455

**Published:** 2025-05-22

**Authors:** Martin Bua Rønhovde, Catia Monteiro, David Dolan, Kjersti Sjøtun, Håkon Dahle

**Affiliations:** ^1^ Department of Biological Sciences University of Bergen Bergen Norway; ^2^ Blue Planet AS Stavanger Norway; ^3^ BIOPOLIS Program in Genomics, Biodiversity and Land Planning Vairão Portugal; ^4^ CIBIO, Centro de Investigação em Biodiversidade e Recursos Genéticos, InBIO Laboratório Associado Vairão Portugal; ^5^ Computational Biology Unit, Department of Biological Science University of Bergen Bergen Norway

**Keywords:** brown algae, gene expression, geographical variation, kelp, population genetics, RNA‐seq

## Abstract

Sugar kelp (*Saccharina latissima*) is an ecologically and increasingly economically important kelp, distributed from temperate to Arctic rocky shores. However, *S. latissima* is presently threatened by ongoing climate changes. Genetic variations have previously been identified across *S. latissima* populations. However, little is known regarding the genetic basis for adaptation and acclimation to different environmental conditions. In this study, a common garden experiment was performed with sporophytes originated from North‐Norway (NN), Mid‐Norway (MN), and South‐Norway (SN), representing areas with highly different temperatures and photoperiods. Transcriptomic analyses revealed significant variation in the gene expression of cultures from North‐Norway, associated with low temperature and long photoperiods, compared to Mid‐ and South‐Norway. Differentially expressed genes included genes linked to photosynthesis, chlorophyll biosynthesis, and heat response, suggesting that they are directly involved in temperature and light adaptation. In addition, genes related to growth, metabolism, protein synthesis, and translation were upregulated in the NN genotype, providing evidence that the NN genotype is better adapted to low temperatures than the SN and MN genotypes. Significant variation in gene expression among populations found in this study is influenced by the environment, but genetic differentiation by origin seems to play a role as responses were population specific. This study provides a baseline for deeper insight into the local adaptation potential of *S. latissima* populations along the Norwegian Coast with implications for the conservation of natural populations.

## Graphical abstract

In this paper, we show that temperature and photoperiod affect the gene expression of sporophytes of *Saccharina latissima* (sugar kelp). Moreover, different populations of *S. latissima* seem to respond differently to variations in temperature and photoperiod, indicating that different populations are specifically adapted to their respective in situ conditions.

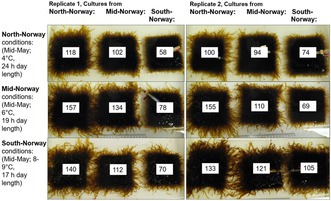

## 1 Introduction

The kelp *Saccharina latissima* is distributed in the temperate and Arctic zones of the North Atlantic and North Pacific (Lüning 1991). In Europe, it forms important habitats, providing shelter for a high diversity of associated fauna (Bekkby et al. 2023). Recent phylogenetic analyses provide evidence that *S. latissima* is composed of four genetically distinct phylogroups, one being the European *S. latissima*, which has also been found on Svalbard (Neiva et al. 2018). In addition, population genetic studies using microsatellites have demonstrated a finer resolution of the genetic structure of *S. latissima* subgroups growing in Europe (Guzinski et al. 2016; Luttikhuizen et al. 2018), with two genetically differentiated groups (northern and southern) identified by Neiva et al. (2018). In Norway, a population genetic study using microsatellites demonstrated good connectivity and little genetic structure in *S. latissima* obtained from South‐Norway, while samples in North‐Norway formed one genetic cluster (Ribeiro et al. 2022).

Being an Arctic‐temperate species, *S. latissima* tolerates sea temperatures from below 0°C to around 20°C, depending on exposure time and life cycle stage (Diehl et al. 2023). *S. latissima* is perennial, and studies have shown a pronounced seasonal cycle, with sporophyte growth normally starting early in the year and peaking in April–June around 60° N (Nielsen et al. 2022). Further, even though there are strong local variations in growth rates (Forbord et al. 2020), there is a tendency of a decrease in the seasonal maximum growth rate with increasing latitude. The peak of the seasonal growth rate is in addition shifted towards June–July in the highest latitudes (Nielsen et al. 2022). *S. latissima* has a broad optimum temperature for growth between 10°C and 15°C (Bolton and Lüning 1982). However, both thermoacclimation or adaptation (Davison et al. 1991; Andersen et al. 2013) and photoacclimation (e.g., Gerard 1988; Davison 1987) have been demonstrated in *S. latissima*. This suggests a strong ability of *S. latissima* to acclimatize or adapt to variable environmental conditions along a latitudinal cline.

One commonly used method to assess acclimation and adaptation in a species is through transcriptomic analysis (Stark et al. 2019). The advantage of transcriptomic analysis over other parameters, such as growth rate, is that gene regulation is often far more sensitive to changes in environmental conditions than physiological parameters. In *S. latissima*, the gene regulation under a range of different environmental conditions has been studied through a series of transcriptomic analyses in *S. latissima* from Svalbard (e.g., Heinrich et al. 2012, 2015, 2016; Li, Scheschonk, et al. 2020; Li, Monteiro, et al. 2020; Monteiro, Li, et al. 2019). Monteiro, Li, et al. (2019) compared material of *S. latissima* originating from Svalbard with material from Roscoff (France), and their results strongly suggested that there were large differences between the two cultures of different origin in gene regulations. However, Svalbard and Roscoff represent extremely distant populations, and transcriptomic responses of *S. latissima* on a smaller geographical scale could give more information on local acclimatization and adaptation. *S. latissima* is present along the Norwegian coast spanning a latitudinal gradient of nearly 27°. Along this gradient, there are strong variations in light availability and temperatures, and physiological and ecological data are abundant for this area. Recent research has shown an overall and concerning trend of decreasing distribution and abundance of *S. latissima* in Norway (Diehl et al. 2023), while the species is in addition targeted for aquaculture in the same area (Sæther et al. 2024). Hence, a better understanding of the patterns underlying species success (or not) is urgently needed.

The overall aim of the study is to analyze differences in gene expression in the kelp *S. latissima* from three different geographical areas exposed to different conditions of temperature and light characteristics in a North–South gradient in Norway during late spring, performed as a common‐garden experiment. We hypothesized that both temperature and photoperiod variation have a large effect on sugar kelp's transcriptomic profiles and that mechanisms related to growth and photosynthesis would be induced more strongly under longer photoperiods and higher temperatures. However, we also expected that distinct *S. latissima* populations have adapted to their respective in situ conditions, and thus their response to changes in temperature and light availability conditions would vary at the transcriptome level.

## 2 Material and Methods

### 2.1 The Common Garden Experiment

A common garden experiment (CGE) was carried out between February 2 and March 13, 2018 at the University of Bergen, with material of *S. latissima* collected in North‐Norway (69°38′ N, 18°57′ E) (NN), Mid‐Norway (63°43′ N, 8°49′ E) (MN), and South‐Norway (60°16′ N, 5°13′ E) (SN). Sea temperature (measured at 4–5 m depth) show strong seasonal variations along the coast of Norway, and differences between the areas of the study sites. In the area of the NN site the average sea temperature varies from a minimum of 2.8°C and 3.0°C in March, to a maximum of 9.4 °C and 10.5°C in August (Sætre 1973; Eilertsen and Skarðhamar 2006). Average measurements from areas close to the MN and SN sites vary from minimum of 4.8°C and 4.0°C, respectively in February/March, to maximum values of 12.9°C and 15.5°C in August (Sætre 1973). Mean annual irradiation (land) also show substantial variation along the coast, and with average values of 799 kWh m^−2^ year^−1^ close to SN, 757 kWh m^−2^ year^−1^ close to MN and 654 kWh m^−2^ year^−1^ close to NN (Riise et al. 2024). However, seasonal day length variations have the highest influence on the light regime along the coast. These are most extreme at the NN site with the polar night lasting from the end November to the middle of January, and the presence of midnight sun between mid‐May and end of July.

Tissue pieces carrying mature sori with sporangia were cut from 10 to 12 *S. latissima*, collected at 1–5 m depth between January 8 and January 12, 2018 at all three sites in Norway. The material from NN and MN was wrapped up in moist paper with cooling elements and transported to the laboratory and seeded the day of arrival, during two successive days. The tissue with sori was treated as described in Forbord et al. (2018) to prevent diatom growth in the cultures, and thereafter submerged in beaker glasses with cool (12°C) sterile sea water and stirred until spore release was observed using a microscope. The spore solution was applied to tagged, clean and heat‐treated granite stones (10 × 10 cm). After 10 min exposure to spore solution the stones were placed in sterile sea water. The material from SN was kept in moist paper in a fridge overnight to provoke spore release and was otherwise treated as described by Forbord et al. (2018). Additional plates for checking development of each culture were seeded in the same way.

The seeded stones were transferred to a climate room. Each batch of gametophytic culture (NN, MN, and SN) was grown in separate tanks with running sea water but with similar photon fluence rates (around 50 μE m^−2^ s^−1^, measured with a spherical sensor) and temperature (9°C–10°C) conditions. The running sea water of the climate rooms is from 100 m depth, filtered through a sand filter, and treated with UV light. The development of each culture batch was checked regularly, and around 20 days after upstart of the cultures minute sporophytes were observed in all three batches.

The CGE was carried out in a climate room divided into three compartments through complete enclosures of black and opaque plastic, where each compartment had two tanks (30 cm × 50 cm × 25 cm [height]) with running seawater, representing replicates (Figure S1). The conditions of the three compartments were set to mimic temperature and day‐length conditions of NN, MN, and SN in mid‐May: NN with 4°C and 24 h light, MN with 6°C and 19 h light, and SN with 9°C and 17 h light. The temperature conditions are within the ranges measured in April–May in the three regions by Forbord et al. (2020) or in mid‐May according to Sætre (1973), which represent a period of rapid growth of *S. latissima*. The granite stones with growing sporophytes were added to the tanks with three stones in each, representing NN, MN, and SN genotypes (Figure S1). The flow rate of running seawater was about 1 L per 20 s in all the tanks, and photon fluence rates were kept at similar levels (46–50 μE m^−2^ s^−1^). The tanks were rinsed regularly to prevent diatom growth, and temperatures of the tanks were checked every second or third day and adjusted when needed. Small variations in temperature, normally within 1°C, occurred during the experiment. On termination, the stones were photographed, and two or three of the largest sporophytes were sampled as parallels per stone, with two stones being replicates for each environmental condition. The samples of sporophytes were put on RNA‐later in a fridge overnight and frozen at −80°C.

Metadata from the common garden experiment is available in Rønhovde et al. (2024).

Using ImageJ, the outline of the kelp covering area of each stone was traced, and total area calculated. This procedure was done twice per stone and the average used. All the stones had a surface of 100 cm^2^, which was extracted from the calculated measurements. The estimated kelp cover exceeding 100 cm^2^ was used as a proxy for kelp growth on each stone.

### 2.2 RNA‐Seq Library/Dataset

Total RNA was extracted from the flash frozen kelp tissue using 2% CTAB and 2M DTT, followed by separation of DNA/RNA from lipids and proteins using chloroform:isoamyl alcohol (24:1). The supernatant was removed, precipitated using isopropanol, pelleted, and washed with absolute alcohol, and finally resuspended in Low TE buffer. Isolation of RNA from extractions was achieved by removing DNA using the TURBO DNA‐free Kit (Invitrogen). Extracted RNA quality and quantity were checked using a Qubit RNA HS kit and Agilent RNA HS kit on a 4200 TapeStation System. RNA integrity numbers (RIN) were in the range of 2.6–6.7, indicating high RNA degradation in all samples. Nevertheless, RNA was sequenced from all RNA extractions with a yield above 500 ng RNA and a concentration of more than 10 ng RNA per μL.

RNA libraries were constructed using the NEBNext Ultra II RNA Library Prep Kit for Illumina (New England Biolabs) and quality checked using the Agilent 4200 TapeStation System. Paired‐end sequencing (2 × 75 bp) of transcriptomes was performed on an Illumina HiSeq4000, giving ca. 30 million reads per sample.

RNA‐seq data was available from 35 samples. Between 1 and 3 sporophytes per stone were analyzed. Within the RNA‐seq subset, sample replicates were categorized according to genotype and temperature/light conditions. Specifically, for the SN genotype, there were four samples each at 6°C/19 h and 4°C/24 h, along with three reference samples at 9°C/17 h. For the MN genotype, the sample distribution was three samples at 9°C/17 h, five samples at 4°C/24 h, and five reference samples at 6°C/19 h. For the NN genotype, there were three reference samples at 4°C/24 h, four samples at 6°C/19 h, and four samples at 9°C/17 h. Transcripts were aligned to a reference genome obtained through the France Génomique National infrastructure project Phaeoexplorer (ANR‐10‐INBS‐09).

### 2.3 Bioinformatics Analysis

Scripts for bioinformatics analyses are available in Rønhovde et al. (2024).

To assess the quality of the raw sequencing reads, the quality control software FastQC (version 0.11.9) was used. The reads were trimmed using Trim Galore (version 0.6.6). Minimum read length before cutoff were set to 60 bp since shorter reads often have low qualities (Krueger 2015). Trim Galore was run with default setting, that is, a Phredscore threshold of 20 and a maximum error rate of 0.1 to obtain clean reads of high quality. MultiQC was used to summarize the results from the read quality trimming done with Trim Galore (Ewels et al. 2016). Trimmed reads were aligned to the reference genome of *S. latissima* using STAR (version 2.7.10a) (Dobin and Gingeras 2015; Denoeud et al. 2024). Prior to alignment, the reference genome was indexed by running ‐‐runmode genomeGenerate. No Gene Transer Format (GTF) file was available and the provided General Feature Format (GFF) file was formatted in STAR to avoid problems when running sequence alignment. This was solved by replacing ‐‐sjdbGTFfile with ‐‐sjdbGTFtagExonParentTranscript. The setting instructs STAR to use “Parent” tag in the GFF‐file to link exons to transcripts. The featureCounts software (version 2.0.1) was used to count the number of reads mapping to each gene in the *S. latissima* reference genome. Pairwise comparisons of differential gene expression were performed using DESeq2 (version 1.34.0) (Love et al. 2014). Reference replicates were compared against replicate groups with same genotype under different conditions. An absolute LFC (log2FoldChange) of ≥ 2 and a *p*‐value of < 0.05 was used as thresholds to determine the significance in gene expression between groups. The correction of *p*‐values in this analysis was handled using the Benjamini–Hochberg procedure, which adjusts for multiple testing by controlling the false discovery rate (FDR). The identifier from the GFF‐file was used to find annotation from Interproscan. A GO enrichment analysis was carried out using topGO (version 2.46.0) Bioconductor package to identify overrepresented GO‐terms. Using the showSigNodes function in TopGO, node trees were generated for the cellular component, biological process, and molecular function categories for NN at 9 °C with a 17‐h light treatment. The figures were edited to enhance clarity and readability.

## 3 Results

### 3.1 Condition of Sporophytes at the End of the Common Garden Experiment

**FIGURE 1:**
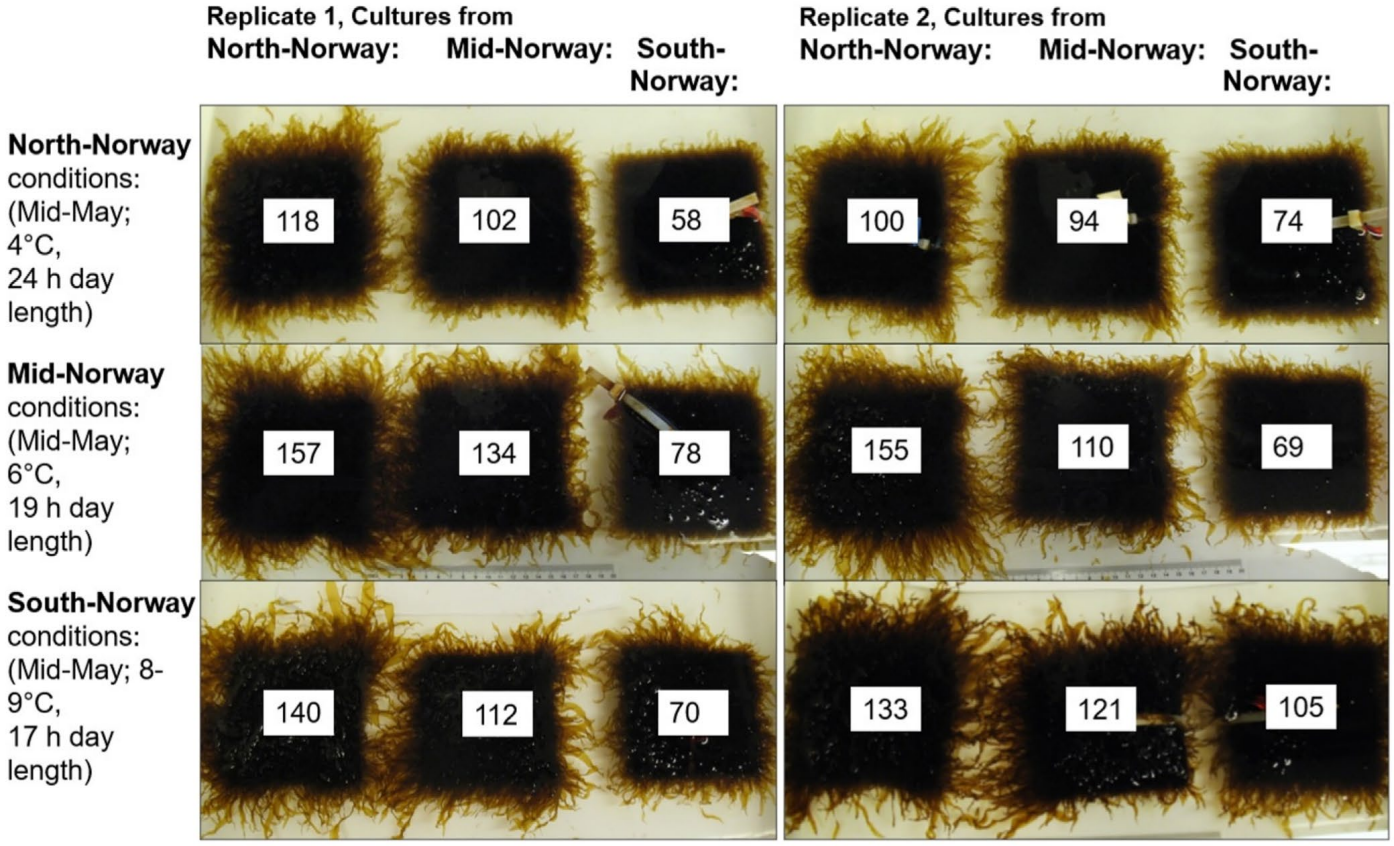
Stones with cultures from NN, MN, and SN grown under NN, MN, and SN conditions are displayed together. The two sets of panels represent replicates. Figures for estimated area of kelp growth (cm^2^) are added to each stone, calculated as total area covered with kelp minus the surface area of each stone (100 cm^2^).

By visual inspection we observed that at the end of the Common Garden Experiment (CGE) the density of sporophytes on the granite stones was high (Figure 1). The length of sporophytes varied considerably, with the longest sporophytes and overall highest estimated growth produced by the NN genotype (Figure 1). Estimates of sporophytic growth suggested that all genotypes grew overall well in SN conditions, whereas the SN genotype grew relatively poorly in NN conditions (Figure 1).

### 3.2 Quality Filtering of Reads

Sequence counts ranged from 29 to 59 million reads per sample. During quality trimming, less than 2% of the reads were removed. Only two samples had sequence counts of less than 30 million after trimming. Moreover, the mean sequencing quality score (Phred) was higher than 38, indicating a low probability for incorrect base calls (Krueger 2015).

### 3.3 Variation in Gene Expression

**FIGURE 2:**
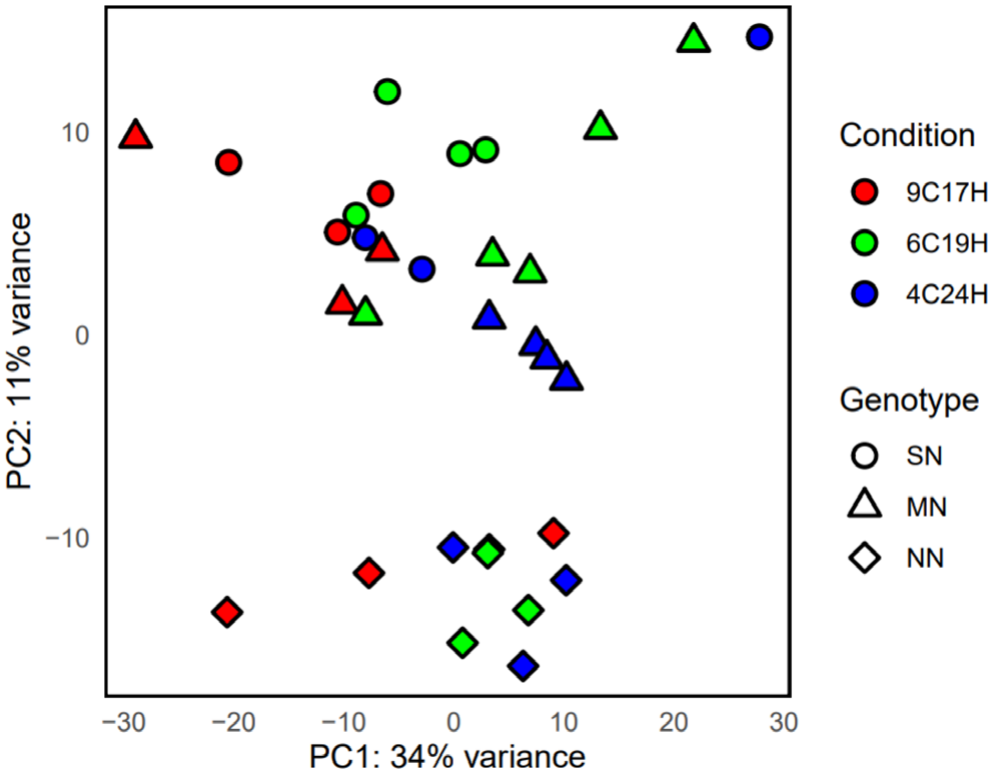
PCA plot based on gene expression profiles of individual samples. South‐Norway = circle, Mid‐Noway = triangle, North‐Norway = diamond, 9°C/17 h light = red, 6°C/19 h light = green, and 4°C/24 h light = blue.

PCA analyses revealed that the geographical origin of the cultures (genotype) and growth conditions drive differential gene expression (Figure 2). The first axis explained 21% of variance and separated samples grown under South‐Norway conditions (9°C/17 h) from samples grown under Mid‐Norway (6°C/19 h) and North‐Norway conditions (4°C/24 h). Samples from SN and MN grouped together, especially for the condition 9°C/17 h. Samples of the NN genotype clearly separated from samples of MN and SN genotypes along the second axis, which explained 13% of the variance, while MN and SN samples grouped together. The spread of replicates within the genotypes revealed variability among samples, which was highest in the SN and MN genotypes, but was also apparent for the NN genotype.

### 3.4 Analysis of Differential Gene Expression

**FIGURE 3:**
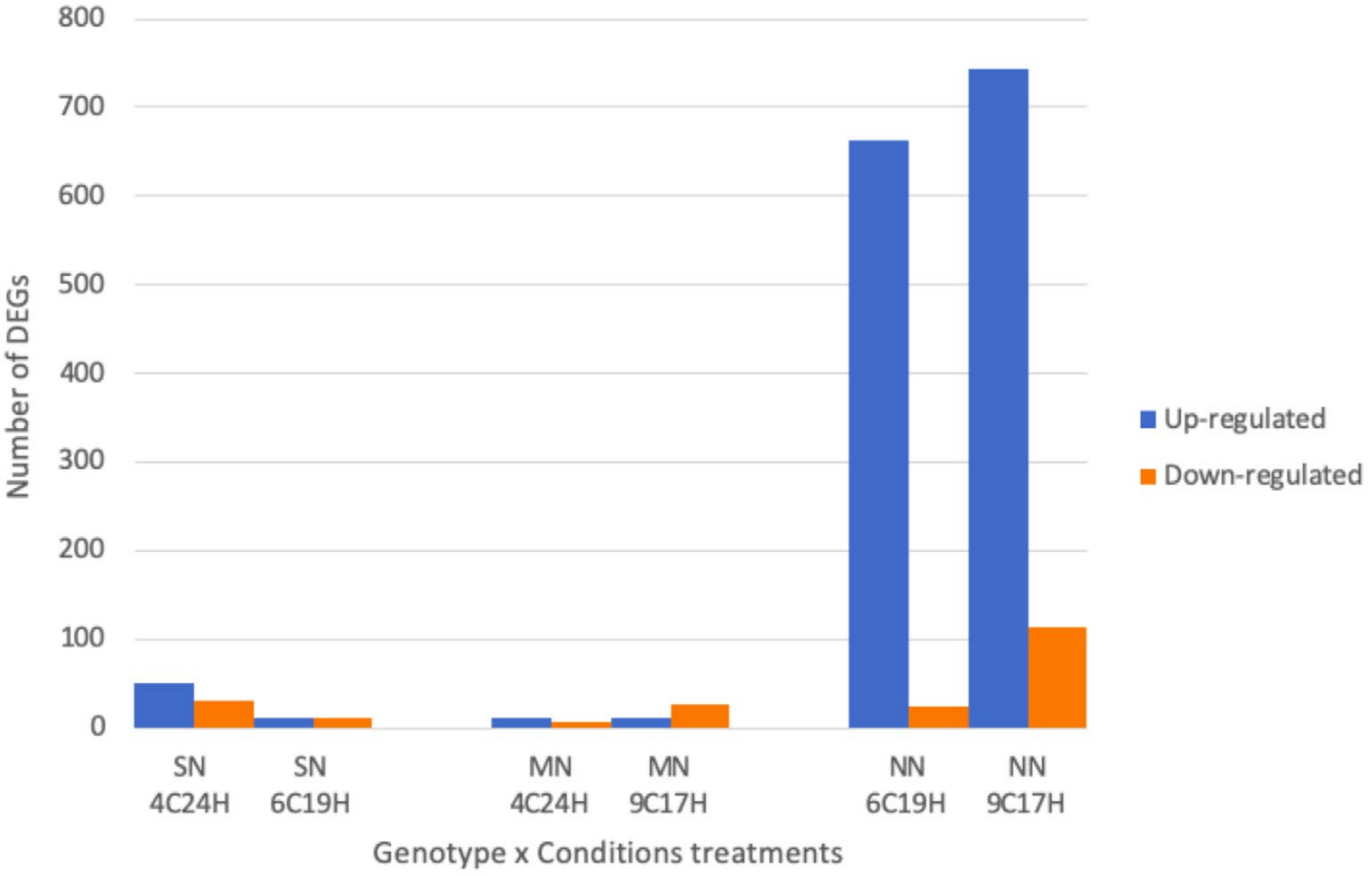
Distribution of differentially upregulated (blue bars) and downregulated (orange bars) genes for sporophytes originated in SN = South‐Norway, MN = Mid‐Norway, and NN = North‐Norway and exposed to different sets of temperature and light conditions (4°C 24 h light: North‐Norway conditions/6°C 19 h light: Mid‐Norway conditions/9°C 17 h light: South‐Norway). Identification of DEGs is based on the Wald test against a control group using a log_2_FoldChange value of > 2 for upregulated genes and < −2 for downregulated genes with a *p*adj‐value and false discovery rate (FDR) of < 0.05.

We identified differentially expressed genes (DEGs) in sample groups exposed to different sets of temperature and day lengths. There were in total 1701 DEGs across all group comparisons with an absolute logFoldChange (FC) value of 2 and *p*‐value < 0.05 (Figure 3). The complete list of the DEGs with a pfam annotation is available in Supporting Information (Tables S1–S3). Overall, the vast majority of DEGs were expressed in the sugar kelp originating from North‐Norway compared to the two other geographical areas. Of the total 1701 DGEs 1544 were found in the NN genotype under MN (6°C/19 h) and SN (9°C/17 h) conditions. Some difference in gene regulation between the two conditions was found, with 168 more DEGs in the samples exposed to 9°C/17 h compared to 6°C/19 h. Moreover, only a small fraction of the total DEGs was expressed in sporophytes from SN and MN. For sporophytes from SN 81 and 23 DEGs were found respectively expressed under 4°C/24 h and 6°C/19 h. The lowest amount of DEGs was found in kelp from Mid‐Norway, with 37 and 16 DEGs expressed under 9°C/17 h and 6°C/19 h. Of the DEGs expressed in NN sporophytes, 91% were upregulated and 11% downregulated under SN conditions (9°C/17 h) compared to the condition of origin (4°C/24 h). For the SN genotype, there were also more upregulated than downregulated genes under the different growth conditions, while for MN the trend was reversed when exposed to 9°C/17 h conditions. Among the most highly expressed DEGs, a few transcripts reached FC of 20. Of the most highly expressed DEGs, 99, 6, 14 DEGs reached an absolute FC value over 5 in samples from NN, MN, and SN, respectively. These results indicate that not only did kelps from NN express more genes (Figure 3), they also expressed them at high levels (Table S1).

### 3.5 DEGs Related to Photosynthesis

A large number of the DEGs were associated with photosynthetic activity (Table 1). In total, seven transcripts related to photosynthetic components were induced in response to treatments with higher temperature and shorter photoperiods for the NN and MN groups. The transcripts encoding photosynthetic components with the highest expression values were found in the NN genotype at SN (9°C/17 h) and MN (6°C/19 h) conditions, and for the MN genotype at SN (9°C/17 h) conditions. Conversely, downregulation of transcripts was observed in the SN genotype at lower temperatures and longer photoperiods (4°C 24 h/6°C 17 h). The gene encoding heat shock proteins hsp 70 family signature was downregulated in the SN genotype at both 6°C/19 h and 4°C/24 h, with a FC of −3.42 and −2.8, respectively. Genes encoding heat shock proteins were not found to be up or downregulated in any of the other genotypes. In addition to transcripts encoding photosynthetic components, we found some DEGs related to Porphyrin and chlorophyll metabolism (Table S6).

**TABLE 1:** Differentially expressed DEGs encoding photosynthetic components.

Contig name	Gene product	NN 9C17H	NN 6C19H	MN 9C17H	MN 4C24H	SN 6C19H	SN 4C24H
Photosynthetic components							
contig3391.10138.1	Photosystem I reaction cenetr subunit IV	**6.25**	**5.43**	**6.70**	NS	−0.14	**−2.88**
contig3385.10112.1	Photosystem I assembly protein	**7.70**	**7.80**	**4.04**	NS	−1.98	**−3.05**
contig3385.10115.1	Photosystem II protein D1	**3.20**	**3.10**	1.44	NS	−0.09	−0.06
contig3953.11043.1	Photosystem II reaction center protein I	**8.64**	**7.27**	**6.38**	NS	−2.33	**−4.71**
contig4255.11547.1	Cytochrome c6/c‐oxidase	**5.56**	**4.89**	**3.04**	NS	−0.39	−1.66
contig2461.7726.1	Photosystem II CP47 reaction center protein	**3.21**	**2.20**	1.41	NS	−0.27	0.39
contig2822.8815.1	Cytochrome b6‐f complex subunit 4	**4.06**	**4.51**	**2.16**	NS	−0.17	0.13

*Note:* NN = North‐Norway, MN = Mid‐Norway, SN = South‐Norway reference replicates under different temperature and photoperiod conditions. Numbers refer to the log fold change (LFC) values of differentially expressed genes (DEGs). Listed genes with numbers in bold are differentially expressed LFC > 2. All genes have a *p*adj‐value of < 0.05. NS = not significant because of insufficient *p*adj‐value to be considered correct and high chance of false discovery (FDR).

### 3.6 DEGs Related to Nucleic Acid Metabolism and ATP‐Synthesis

DEGs associated with nucleic acid metabolism were identified among the most DEGs. Replicative DNA helicase (contig2461.7724.1) was the most highly expressed transcript with a FC value of 8.58 and 7.40 for the NN group at 9°C/17 h light and 6°C/19 h light, respectively (Table 2). Genes encoding tRNA synthetase were also detected for the NN genotype. Conversely, these transcripts were not differentially expressed in the MN or SN genotypes at any condition. Interestingly, Reverse transcriptase (RNA‐dependent DNA polymerase) (contig4003.11142.1) was found highly upregulated in the MN and SN genotypes at 9°C/17 h and 4°C/24 h, respectively, with a FC of 15.7 and 22.7. Reverse transcriptase was the most DEG for the SN genotype at 4°C/24 h and the second most expressed in the MN genotype at 9°C/17 h (Tables S2 and S3). Rieske [2Fe‐2S] domain in the MN genotype at 4°C/24 h emerged as the highest scoring DEG of all.

**TABLE 2:** DEGs related to nucleic acid metabolism.

Contig name	Gene product	NN 9C17H	NN 6C19H	MN 9C17H	MN 4C24H	SN 6C19H	SN 4C24H
Nucleic acid metabolism							
contig2461.7724.1	Replicative DNA helicase	**8.58**	**7.40**	0	0	0	0
contig2461.7722.1	tRNA synthetase	**7.9**	**5.99**	0	0	0	0
contig2461.7722.1	Phenylalanyl tRNA synthetase beta chain CLM domain	**7.90**	**5.99**	0	0	0	0
contig4003.11142.1	Reverse transcriptase (RNA‐dependent DNA polymerase)	0	0	**15.7**	0	0	**22.7**

*Note:* NN = North‐Norway, MN = Mid‐Norway, SN = South‐Norway reference replicates under different temperature and photoperiod conditions. Numbers refer to the log fold change (LFC) values of differentially expressed genes (DEGs). Listed genes with numbers in bold are differentially expressed LFC > 2. All genes have a *p*adj‐value of < 0.05.

### 3.7 DEGs Related to Protein Metabolism, Proteases and Ribosomal Genes

Transcripts encoding for ribosomal genes were among the most abundant and highly expressed DEGs in NN at 6°C/19 h and 9°C/17 h (Table 3, Table S7). From the top 20 differentially expressed transcripts, 6 out of 20 transcripts were genes encoding for ribosomal proteins. A total of 13 upregulated transcripts were detected after manual inspection of the gene annotation text file for the pairwise comparisons in NN at conditions with 9°C/17 h light and 6°C/19 h light (Table 3). Most transcripts (8 out of 13) responded most strongly at 9°C/17 h light in the NN genotype, although some transcripts were more highly expressed at 6°C/19 h. Expression levels expressed by LFC ranged from 4.32 to 8.29, peaking for Ribosomal protein S2 signature (contig282288091) in NN genotype at 9°C/17 h conditions. No ribosomal proteins were expressed in the SN genotype at either of the two conditions, while Ribosomal protein S2 signature (contig282288091) and Ribosomal protein L2 signature (contig2461.7736.1) were differentially expressed in the MN genotype at 9°C/17 h conditions. Magnesium chelatase ATPase was also expressed at high levels in the NN genotype at 9°C/17 h and 6°C/19 h with FC of 4.43 and 6.20, respectively, and in the MN genotype at 9°C/17 with a FC of 3.76. Thus, Magnesium chelatase ATPase was expressed at high levels in genotypes with temperature conditions higher than its origin.

**TABLE 3:** DEGs related to ribosomal proteins.

Contig	Gene product	NN 9C17H	NN 6C19H	MN 4C24H	MN 9C17H	SN 4C24H	SN 6C19H
contig2822.8809.1	Ribosomal protein S2 signature	**8.29**	**6.36**	NS	**5.40**	NS	NS
contig2461.7725.1	Ribosomal protein L1p/L10e	**8.05**	**6.84**	NS	NS	NS	NS
contig2822.8818.1	30S ribosomal protein S16	**7.85**	**6.28**	NS	NS	NS	NS
contig2461.7723.1	Ribosomal protein L9	**7.27**	**6.68**	NS	NS	NS	NS
contig2822.8819.1	30S ribosomal protein S4	**7.06**	**7.36**	NS	NS	NS	NS
contig1308.2337.1	Ribosomal protein S7 signature	**5.95**	NS	NS	NS	NS	NS
contig238.7448.1	30S ribosomal protein S14 type Z	**5.64**	**5.52**	NS	NS	NS	NS
contig189.5420.1	Ribosomal L39 protein	**5.62**	**5.41**	NS	NS	NS	NS
contig2461.7736.1	Ribosomal protein L2 signature	**5.49**	**4.47**	NS	**3.95**	NS	NS
contig4328.11696.1	Ribosomal protein L21 signature	**5.48**	**6.18**	NS	NS	NS	NS
contig2461.7735.1	Ribosomal protein L16 signature	**5.19**	**4.45**	NS	NS	NS	NS
contig2461.7728.1	30S ribosomal protein S12	**5.06**	**4.65**	NS	NS	NS	NS
contig3391.10135.1	30S ribosomal protein S7	**4.97**	**4.32**	NS	NS	NS	NS

*Note:* NN = North‐Norway, MN = Mid‐Norway, SN = South‐Norway reference replicates under different temperature and photoperiod conditions. Numbers refer to the log fold change (LFC) values of differentially expressed genes (DEGs). Listed genes with numbers in bold are differentially expressed LFC > 2. All genes have a *p*adj‐value of < 0.05.

Several highly expressed transcripts encoding proteases were found in the NN genotype under MN and SN conditions (Table 4). ATP‐dependent Clp protease ATP‐binding subunit (contig3385191131) was the most differentially expressed transcript with an LFC value of 7.40 and 7.10 for the 6°C/19 h light and 9°C/17 h light conditions, respectively (Table S8). The presence of DEGs encoding proteases could indicate that there is activity related to the breakdown of proteins and signaling when the NN group experiences changes in temperature and photoperiod. Proteases are also shown to produce new protein products and influence transcription and cell differentiation (López‐Otín and Bond 2008). Moreover, none of the proteases were found at significant levels in either MN or SN at any given condition (Table S3).

**TABLE 4:** DEGs related to proteases.

Contig name	Gene product	NN 9C17H	NN 6C19H	MN 4C24H	MN 9C17H	SN 4C24H	SN.6C19H
contig1340.2532.1	CPBP intramembrane metalloprotease	**2.80**	**2.60**	NS	NS	NS	NS
contig26.8124.1	Clp protease catalytic subunit P	1.90	1.30	NS	NS	NS	NS
contig370.10639.1	ATP‐dependent Clp protease adaptor protein	**2.60**	**2.30**	NS	NS	NS	NS
contig3385.10113.1	ATP‐dependent Clp protease ATP‐binding subunit	**7.40**	**7.10**	NS	NS	NS	NS
contig209.6322.1	ATP‐dependent protease HslVU, peptidase subunit	**2.50**	**2.20**	NS	NS	NS	NS
contig606.13893.1	OTU‐like cysteine protease	**2.20**	**2.00**	NS	NS	NS	NS
contig426.11559.1	Ubiquitin protease	**2.30**	**2.40**	NS	NS	NS	NS

*Note:* NN = North‐Norway, MN = Mid‐Norway, SN = South‐Norway reference replicates under different temperature and photoperiod conditions. Numbers refer to the log fold change (LFC) values of differentially expressed genes (DEGs). Listed genes with numbers in bold are differentially expressed LFC > 2. All genes have a *p*adj‐value of < 0.05.

### 3.8 GO Enrichment Analysis

Gene ontology enrichment analysis was conducted to interpret the function and classes of DEGs. Consistent with the overall DEG analysis (Figure 3), most enriched GO terms were found for upregulated transcripts in the NN genotype under 6°C/19 h light and 9°C/17 h light conditions. Only 1 or 2 GO terms were significantly enriched in each category at most in the SN and MN genotypes, whereas the NN genotype had a vast tenfold enrichment in comparison. See Table S9 in Supporting Information for results from the Fisher test for classification of GO‐terms functional groups.

**FIGURE 4:**
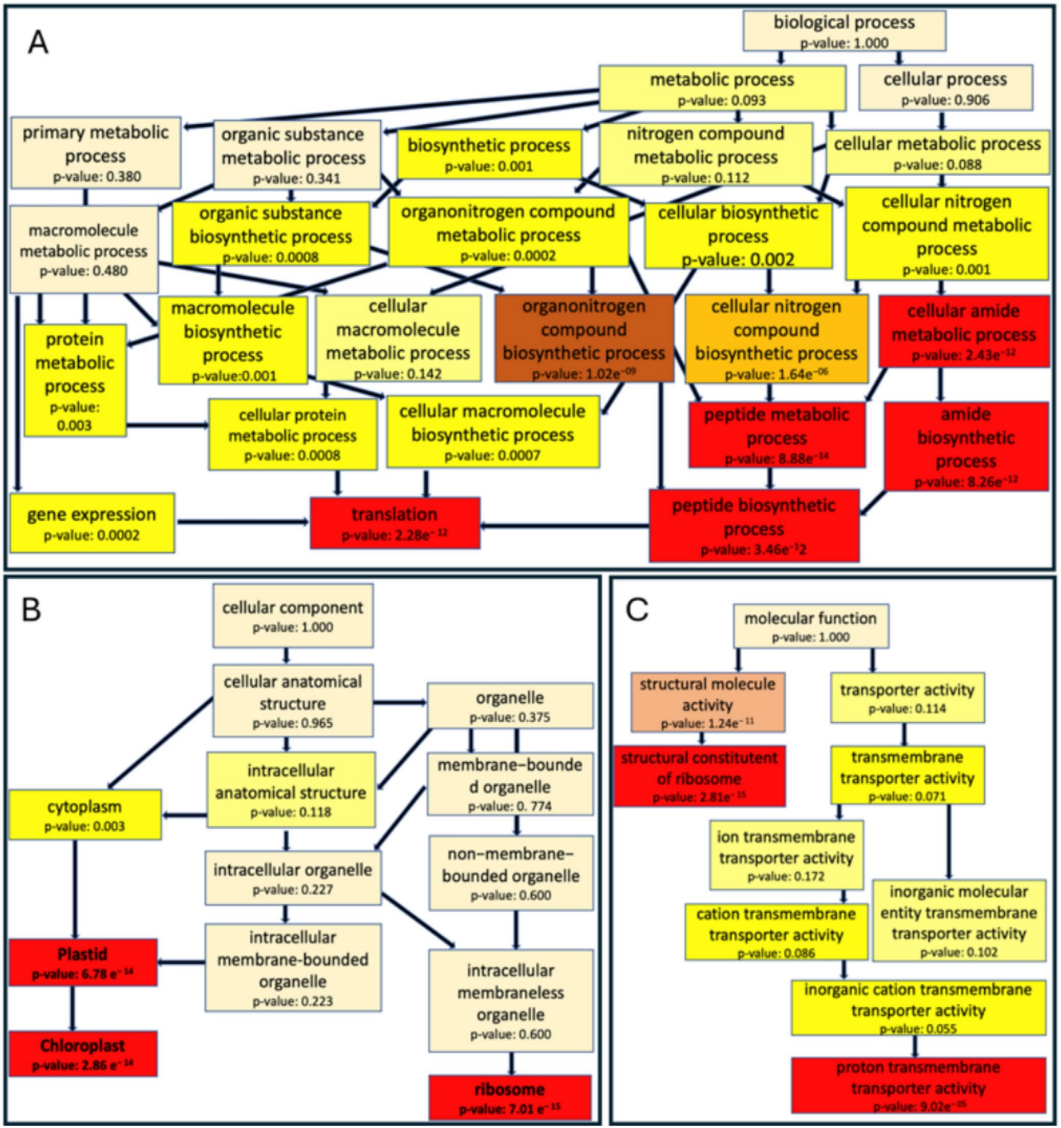
TopGO‐directed acyclic graphs of GO term enrichment for NN at 9°C under 16H light treatment. Panels A, B, and C display the enriched GO nodes for the cellular component, biological process, and molecular function categories, respectively. Each node is depicted as a box that provides a brief description of the GO term product and the corresponding *p*‐value. Connecting lines illustrate the hierarchical relationships among GO terms. Color intensities reflect statistical significance, with deeper shades indicating higher enrichment significance.

GO terms are categorized as cellular component (CC), biological process (BP), or molecular function (MF). In the cellular component category at 9°C/17 h condition for the NN genotype, 1480 genes had a GO term annotation, with 119 genes being significantly enriched (Table S4). Analyses involving 1194 GO terms revealed 61 terms associated with differential expression (*p*‐value < 0.01). Regarding cellular components, ribosome‐related terms were most enriched, followed by chloroplast and plastid terms (Table S4). Top GO terms included peptide metabolic process, translation, and cellular amide metabolic process (Figure 4). Gene expression‐related terms were also prevalent. In the molecular function category, 1248 annotated genes had 97 significantly enriched genes (Table S5). For biological process ontology, 1434 genes were annotated with 107 significant genes and 51 enriched GO terms. Top GO terms included structural constituent of ribosome, structural molecule activity, and proton transmembrane transporter activity (Table S5).

Comparatively, the NN genotype under 6°C/19 h light conditions had similar results, with the same GO terms significantly enriched but slightly fewer significant genes annotating to a GO term (Table S5). The cellular component category had 111 significant genes, biological process had 98, and molecular function had 92. Top GO terms were consistent with those at 9°C/17 h light.

In contrast, GO terms for downregulated genes were less enriched. The NN genotype under 9°C/17 h light condition had 5–6 significant genes in each category, while only 1 was significant in 6°C/19 h light condition. Few to no enriched GO terms were found in the SN and MN genotypes for both condition contrasts, with 1–2 significant genes annotated to a GO term and low scores in all three categories.

## 4 Discussion

*Saccharina latissima* beds contribute to climate change mitigation through efficient carbon capture; they reduce eutrophication by nutrient removal (Duffy et al. 2019) and enhance biodiversity by creating habitats for associated species (Bekkby et al. 2023). Sugar kelp is currently threatened by ongoing warming and anthropogenic pressures; hence, identifying the role of genetic diversity (Thomson et al. 2021) and differential gene expression (Monteiro, Heinrich, et al. 2019) in response to changing environmental factors is crucial for understanding the resilience of species to climate changes.

Furthermore, identifying locally adapted strains within specific geographical areas is essential for sustainable conservation and management of *S. latissima*.

To evaluate how geographical variation modulates responses of *S. latissima*, we conducted transcriptomic analyses on *S. latissima* sporophytes from three Norwegian regions grown under common garden conditions. Our hypothesis posited that distinct *S. latissima* populations have adapted to their respective in situ conditions, and thus their response to changes in temperature and light availability conditions at the transcriptome level is expected to vary. It should be noted that RIN values associated with RNA extraction were low (2.6–6.7) indicating high RNA degradation. How RIN values are connected to sequencing results is unclear (Galligo Romero et al. 2014). There is a possibility that different transcripts are degraded at different rates, potentially biasing expression levels. Our results should therefore be interpreted with some caution. However, we consider it unlikely that such potential biases have a large effect on the major trends we observed regarding what types of genes are differentially expressed and how gene expression profiles vary between populations and environmental conditions.

### 4.1 Geographical Variation in Gene Expression to Temperature and Light

The results revealed a substantial difference in gene expression among the genotypes. The NN genotype showed strong up and downregulation of many genes in MN and SN growth conditions, while the MN and SN genotypes showed, in general, much less variation in gene expression. In addition, the NN genotype developed relatively long sporophytes during the CGE, irrespective of growth conditions. The findings suggest that the NN genotype possesses some unique genetic characteristics compared to the SN and MN genotypes, contributing to a divergent adaptation pattern. This observation aligns with population genetic research of *S. latissima* from Norway, where a distinct North‐Norway genetic group has been found north of the Lofoten archipelago (Evankow et al. 2019; Ribeiro et al. 2022), probably partly because of the Lofoten archipelago acting as a barrier for gene flow northwards, causing some isolation of *S. latissima* north of the archipelago. While distinct genetic clusters may not necessarily indicate different adaptations, an observed genetic differentiation can coincide with local adaptation (Durrant et al. 2018).

While the SN and MN genotypes showed less regulation of gene expression, some were still evident. The SN culture showed, for example, more up and downregulated genes than the MN culture under NN conditions. Ribeiro et al. (2022) did not find specific genetic clusters of *S. latissima* on the coast of South‐Norway, but their results showed a clear isolation by distance pattern, suggesting some genetic differentiation. Evankow et al. (2019) showed that genetic differentiation in *S. latissima* along the Norwegian coast was weaker than for the other commonly habitat‐forming kelp in the area, *Laminaria hyperborea* . Still, three and four clusters were identified for *S. latissima* and *L. hyperborea* , respectively. Similarly, geographical variation in demography and performance of *L. hyperborea* along the Norwegian coast has been reported (Gundersen et al. 2021; Rinde and Sjøtun 2005). Nevertheless, studies such as this exploring geographical variation in species' responses to the differential environment across the Norwegian coastline are rare for coastal organisms, limiting our understanding of the effects of climate change in this coast. Genetic differentiation and local adaptation can persist even under high gene flow rates (Tigano and Friesen 2016). However, the differences in gene expression between SN and MN genotypes of *S. latissima* were very small compared to the difference between the NN genotype and the SN and MN genotypes.

The latitudinal and seasonal variation of sea water temperature and day lengths results in a combination of much light and low temperatures during late spring and summer in polar regions. This combination may cause a cellular energy imbalance in algae, due to the rapid, temperature‐insensitive processes of light absorption being integrated with the slower and temperature‐dependent redox reactions of electron transport and enzyme reactions (Hüner et al. 2022). The results of the present study suggest that the NN genotype is capable of adjusting gene expression and growing well under the condition with the lowest temperature and longest day lengths. However, while NN sporophytes did appear to grow better at 4°C/24 h light (origin condition) than the MN and SN genotypes did, the NN genotype either performed as well or even better than the SN and MN genotypes in their respective environmental conditions. Such a population‐specific temperature response of northern *S. latissima* aligns with findings by Monteiro, Li, et al. (2019), where a stronger modulation of gene expression in sporophytes from Brittany was required than in sporophytes from Spitsbergen to adjust to the Arctic conditions.

Large transcriptomic reprogramming, including inducing photosynthesis and growth related processes in response to higher temperatures, may represent a general adaptation of *S. latissima* in the cold temperate or Arctic region. While laboratory experiments with *S. latissima* have shown a broad optimum temperature for growth at 12°C–15°C, a shift to a lower optimum temperature for growth of around 10°C was found for Arctic *S. latissima* (as *L. longicruris*) (Bolton and Lüning 1982). Also, Diehl and Bischof (2021) found that the size increase of *S. latissima* from Spitsbergen was considerably larger than that of *S. latissima* from Bodø (south of the Lofoten arhipelago) when cultivated at 12°C (confer table 1 and figure 3 in Diehl and Bischof 2021). Considering the exposure of *S. latissima* in North‐Norway to suboptimal temperatures for at least the main part of the year, it could be argued that the population is adapted to be temperature‐sensitive in the low temperature range, responding rapidly to an increase in temperature to enhance growth during spring. Arctic populations of *S. latissima* are dependent on developing a big lamina during spring and early summer for photosynthesis and storage of laminarin, for survival during the Arctic night (Scheschonk et al. 2019).

The distribution and abundance of *S. latissima* in North‐Norway is presently strongly influenced by sea urchin grazing, which has led to a large‐scale decrease in the abundance of kelp in North‐Norway over decades (Norderhaug and Christie 2009). Sea urchin grazing thus poses a threat to the North‐Norway genetic cluster of *S. latissima* in North‐Norway, and the unique genetic features *S. latissima* possesses here may disappear. In light of the results of this study, this could mean loss of an important cold water adapted genetic reservoir. In the case of a subsequent future northward expansion of more southern *S. latissima*, it is not clear if such an influx of *S. latissima* will readily adapt to NN conditions. The combination of increasing sea water temperatures and high latitude light environment with polar night will probably be challenging for Arctic *S. latissima* (Scheschonk et al. 2019; Li, Scheschonk, et al. 2020), and future adaptation of different genetic groups of *S. latissima* to the climate changes of the Arctic should be given more attention.

Determining with certainty which conditions a population is adapted to remains challenging. Also, the potential overestimation of transcriptomic responses due to laboratory‐cultivated sporophytes displaying more gene regulation than field sporophytes of *S. latissima* (Heinrich et al. 2016) warrants consideration. In addition to light and temperature, other factors like salinity, nutrients, and turbidity will influence photosynthesis activity and growth in *S. latissima*. Such factors have been associated with genetic markers in *S. latissima* (Guzinski et al. 2020). The present study focused solely on different temperatures and day lengths, while other factors were kept stable and not limiting for growth. This makes it difficult to draw definitive conclusions about local adaptation. However, hyposalinity has been shown to lead to strong downregulation of several metabolic pathways in sporophytes of *S. latissima* (Monteiro, Li, et al. 2019; Li, Monteiro, et al. 2020). In another study, in response to the combined effects of warming, hyposalinity, and lower irradiance, genes related to cytoskeleton, transcription/translation, and metabolism, among others, were downregulated in *S. latissima* (Lebrun et al. 2024). However, the authors found that warming alone had the strongest effect on gene expression, which does not agree with the previous study, where salinity variation led to a stronger response than warming (Monteiro, Li, et al. 2019; Li, Monteiro et al. 2020). This might be due to differences in levels tested and/or the origin and age of sporophytes, which highlights the need for more studies on the topic. Future research should also explore a broader range of temperatures and other environmental factors to enhance understanding of local adaptation in the species. The recent discoveries of epigenetic responses in *S. latissima* (Scheschonk et al. 2023) should also be taken into consideration. In a recent study, the authors found that samples of *S. latissima* from Spitsbergen (Arctic) had a significantly lower number of methylated sites, whereas levels of methylation were higher than in samples from Helgoland (temperate) in both the nucleus and the chloroplast (Scheschonk et al. 2023, 2024). In addition, genes related to photosynthesis were differentially methylated between the two populations. A similar mechanism might be taking place in the populations tested here as, though we employed a common‐garden approach, we cannot exclude the role of environmental history in our samples. Further studies are needed to disentangle the role of genetic adaptation, epigenetics, and phenotypic plasticity in the response of *S. latissima* to abiotic factors.

### 4.2 Differentially Expressed Genes and Gene Ontology in Response to Temperature and Light

The DEGs in the different light and temperature conditions were directly linked to photosynthesis, heat response, and chlorophyll metabolism. Genes encoding Photosystem I and II proteins, Magnesium chelatase ATP‐ase, and heat shock hsp 70 were among those identified. Genes involved in Photosynthetic processes were upregulated in NN at 6°C/19 h light and 9°C/17 h light (Table 1). The upregulation of Magnesium chelatase, a regulatory step in chlorophyll biosynthesis, may be associated with heightened photosynthetic activity in response to increased temperature (Heinrich et al. 2015). Enriched GO‐terms for various biosynthetic processes further supported these findings. Heat shock proteins were differentially expressed in this study even under the low temperature range tested. While HSPs are to some extent associated with high temperature levels, recent findings suggest a more nuanced response than solely to high heat adaptation. As mentioned in Monteiro, Li, et al. (2019), HSPs are often responsive to abiotic stress and can be induced in shorter‐term responses, but their expression may diminish during longer‐term acclimation. This is consistent with the observed discrepancy in the expression of only one HSP in our experiment conducted over a duration of 40 days. Based on this insight, experimental temperature conditions ranging from 4°C to 9°C may not have been high enough to induce a pronounced HSP expression response. At any rate, attributing HSP expression solely to temperature stress warrants caution, and a broader understanding of their regulation in response to diverse environmental factors is crucial.

Strong regulation occured in genes coding for proteases, Rieske‐Domain, GDP‐mannose 4,6 dehydratase, and ribosomal proteins. The expression of protease genes in NN at 6°C and 19 h and 9°C and 17 h suggests a potential role in plastid protein synthesis and quality control as ‘housekeeping genes’ (Sjögren et al. 2006). As a part of adaptation to higher temperatures over time, plastid protein synthesis and quality control may be a part of this process to adjust and maintain proper plastid function. We hypothesize that the upregulation of these genes is associated with precise protein synthesis regulation within the cell, preventing mal‐formations. Additionally, plastid genome‐encoded genes are primarily involved in chloroplast gene expression or photosynthesis (Ahlert et al. 2003). Thus, the differential expression of these genes suggests connection with changes in temperature and light.

The highest‐scoring differentially expressed gene, Rieske [2Fe‐2S] domain, was expressed in MN under 4°C/24 h, indicating a role in electron transport and ATP synthesis (Trumpower 2013; Gabellini and Sebald 1986). Enriched GO terms associated with ATP synthase and ATPase activity in NN, along with gene expression and translation, indicate increased ATP investment for mRNA processing and translation (Monteiro, Heinrich, et al. 2019), which ultimately resulted in increased growth. In summary, sporophytes from NN responded to the conditions of MN and SN (higher temperatures, but lower photoperiod) by inducing genes related to photosynthesis, protein synthesis, and DNA metabolism, all necessary for a higher growth rate. Results indicate that the increase in temperature has a stronger impact on photosynthesis and growth than the photoperiod tested here (24–17 h). This is in agreement with results from former experiments (Lee and Brinkhuis 1988; Egan et al. 1989). This is probably related to the fact that 17 h light is already sufficient for photosynthesis and growth and that the increased metabolic rate with higher temperatures, albeit a small difference, is more relevant here.

Moreover, the upregulation of GDP‐mannose 4,6 dehydratase in the NN genotype under 9°C/17 h can be associated with increased alginate production and cell wall formation in *Saccharina* sp. (Zang et al. 2020), in contrast to the SN genotype under 6°C/19 h conditions where this gene was not upregulated and which showed less growth.

The results could also have broader implications for management and conservational efforts on *S. latissima*. One point to consider is the extensive sea urchin grazing of *S. latissima* in North‐Norway, where large areas have been affected. To efficiently restore areas where kelp has disappeared, the use of “green gravel” has been suggested, which is gravel seeded with kelp and outplanted (Fredriksen et al. 2020). Our results suggest that possible restoration attempts of *S. latissima* using such methods should be carried out using local kelp material. Arguably, populations with more genetic variety and resilience should be conserved as a future “reservoir” for genetic diversity (Guzinski et al. 2020). Hence, the present study highlights the need to protect northern Norway populations of *S. latissima*. However, it needs to be taken into consideration that even though increased temperature seems to be benefiting the northern genotype, other environmental factors changing due to global warming might be detrimental, such as coastal darkening and decreased salinity due to melting of glaciers (Li, Scheschonk, et al. 2020; Frigstad et al. 2023; Blain et al. 2021).

It must be acknowledged that analyses of transcriptomic responses in brown algae are still hampered by the lack of gene functional information, but this is quickly changing. Also, gene expression only looks at components of responses to stimuli. Further work should measure other parameters beyond transcriptomic responses, such as physiological parameters, metabolomics, and epigenetics. Transplant experiments in the field could also help elucidate local adaptation occurring between these populations. Furthermore, comparison with other populations along the distributional range of *S. latissima* (that extends to the north of Portugal in the Atlantic and it is also present in the NW Atlantic) could better explain the role of phenotypic plasticity versus local adaptation in the persistence of the species.

This study contributes valuable insights into the limited knowledge of transcriptomic data on local adaptation in *S. latissima*. The evidence of potentially local adaptation is supported by distinct transcriptomic profiles in NN, higher abundance of differentially expressed genes at specific conditions, and observed phenotypic differences within population cultures in controlled growth environments.

## Author Contributions

**Martin Bua Rønhovde:** formal analysis (equal), investigation (equal), methodology (equal), writing – original draft (lead), writing – review and editing (equal). **Catia Monteiro:** conceptualization (supporting), methodology (supporting), supervision (supporting), writing – review and editing (equal). **David Dolan:** methodology (equal), supervision (supporting), writing – review and editing (equal). **Kjersti Sjøtun:** conceptualization (lead), formal analysis (equal), funding acquisition (lead), methodology (equal), project administration (equal), supervision (lead), writing – original draft (supporting), writing – review and editing (equal). **Håkon Dahle:** formal analysis (supporting), methodology (supporting), supervision (lead), writing – original draft (supporting), writing – review and editing (equal).

## Conflicts of Interest

The authors declare no conflicts of interest.

## Supporting information


Figure S1.

Tables S1–S9.

## Data Availability

RNAseq‐data: Raw sequence reads are deposited in the SRA (BioProject: PRJNA1164979). The reference genome of *S. latissima* is available from the Phaeoexplorer Brown Algal Genome Database (https://phaeoexplorer.sb‐roscoff.fr/organism/saccharina‐latissima_female/). Metadata: Related metadata, including photoperiod, temperature, geo‐references and date/month/year of sampling, can be found in SRA (BioProject: PRJNA1164979), and in DRYAD (https://doi.org/10.5061/dryad.bk3j9kbnb). Bioinformatics pipelines: Bioinformatics pipelines are available from DRYAD/ZENODO (zenodo.org/records/13918980) Benefits generated: Benefits from this research accrue from the sharing of our data and results on public databases as described above.
