# Supplementary material for: Northern Norway Sporophytes of Saccharina latissima Display Distinct Gene Expression Profiles in Response to Temperature and Photoperiod
Source: Ecol Evol. 2025 May 22;15(5):e71455. doi: 10.1002/ece3.71455 (PMC12098305; doi:10.1002/ece3.71455)

**Figure S1**: A schematic overview of the set-up of the common garden experiment. The six large rectangles represent the six tanks of the climate room, and the three blue color shades represents the three environmental conditions of the tanks; dark blue represents the environmental conditions of South-Norway (tank 1 and 2), intermediate blue the environmental conditions of Mid-Norway (tank 3 and 4) and pale blue the environmental conditions of North-Norway (tank 5 and 6). The three small rectangles per tank represent granite stones with juvenile sporophytes of *Saccharina latissima*, where SN is genetic material from South-Norway, MN genetic material from Mid-Norway and NN genetic material from North-Norway.


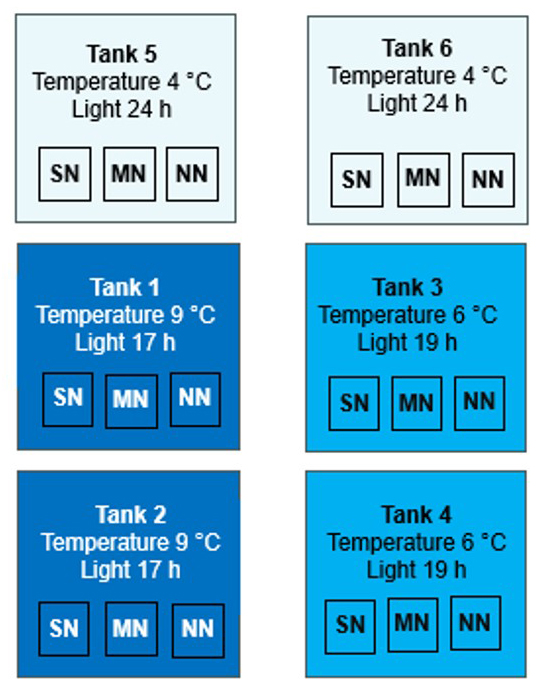

Supplement: Supplementary file 1 — Figure S1. [file ECE3-15-e71455-s001.docx]
