# Supplementary material for: Northern Norway Sporophytes of Saccharina latissima Display Distinct Gene Expression Profiles in Response to Temperature and Photoperiod
Source: Ecol Evol. 2025 May 22;15(5):e71455. doi: 10.1002/ece3.71455 (PMC12098305; doi:10.1002/ece3.71455)
Supplement: Supplementary file 2 — Tables S1–S9. [file ECE3-15-e71455-s002.docx]

**Supplementary Material**

Table S1: Most up/down- regulated DEGs with annotation table based on highest and lowest LFC-value side by side of NN at 6°C 19h light and 9°C 17h light. Genotype, condition and LFC-value is shown in the first three columns. The second and fourth column is the gene product described from the output annotation file from the PFAM database. All genes have a padj value of <0.05.

| **Genotype: North-Norway** | | | |
| --- | --- | --- | --- |
| **Condition 9°C 17h light** |  | **Condition**  **6°C 19h light** |  |
| **LFC-value** | **Gene product** | **LFC-value** | **Gene product** |
| -22.8 | Protein tyrosine kinase | -21.6 | Protein tyrosine kinase |
| 8.6 | Photosystem II reaction centre I protein | 7.8 | Photosystem I assembly protein Ycf3 |
| 8.6 | Replicative DNA helicase | 7.4 | DnaB-like helicase C terminal domain |
| 8.3 | Ribosomal protein S2 | 7.3 | 30S ribosomal protein S4 |
| 8.0 | Ribosomal protein L1 signature | 7.3 | Photosystem II reaction centre I protein |
| 7.9 | Photosystem II reaction centre X protein | 7.1 | S1 RNA binding domain |
| 7.9 | tRNA synthetase | 7.1 | ATP-dependent Clp protease ATP-binding subunit |
| 7.9 | 30S ribosomal protein S16 | 6.8 | Ribosomal protein L1p/L10e family |
| 7.8 | GDP-mannose 4,6 dehydratase | 6.8 | Thiamine pyrophosphate enzyme |
| 7.7 | Photosystem I assembly protein Ycf3 | 6.7 | Ribosomal protein uL1 |
| 7.5 | S1 RNA binding domain | 6.7 | NmrA-like family |
| 7.4 | ATP-dependent Clp protease ATP-binding subunit | 6.5 | ADP-ribosylation factor family |
| 7.4 | Elongation factor Ts signature | 6.4 | Arginase family |
| 7.3 | Thiamine pyrophosphate enzyme | 6.3 | Ribosomal protein S2 |
| 7.3 | Ribosomal protein L9 | 6.3 | 30S ribosomal protein S16 |
| 7.1 | Ribosomal protein S4/S9 | 6.2 | Elongation factor Ts signature |
| 7.0 | NmrA-like family | 6.2 | Magnesium chelatase |
| 6.9 | ADP-ribosylation factor family | 6.2 | Ribosomal protein L21 signature protein S4 |
| 6.72 | AAA lid domain | 6.2 | Photosystem II reaction centre X protein |
| 6.45 | Photosystem I iron-sulfur protein | 6 | Phenylalanyl tRNA synthetase beta chain CLM domain |

Table S2: Top most up/down- regulated DEGs identified by DESeq2 with annotation table based on highest and lowest LFC-value comparing MN at 9°C 17h light and 4°C 24h light. The second and fourth column is the gene product described from the output annotation file from the PFAM database. All genes have a padj value of <0.05.

| **Genotype: Mid-Norway** | | | |
| --- | --- | --- | --- |
| **Condition 9°C 17h light** |  | **Condition**  **4°C 24h light** |  |
| **LFC-value** | **Gene product** | **LFC-value** | **Gene product** |
| 20.9 | N-terminal region of a signal peptide | 23.9 | Rieske [2Fe-2S] domain |
| 15.7 | Reverse transcriptase (RNA-dependent DNA polymerase) | 4.8 | Region of a membrane-bound protein predicted to be outside the membrane, in the extracellular region |
| 6.4 | Photosystem II reaction centre I protein | 3.5 | PAS domain S-box protein |
| 5.4 | Ribosomal protein S2 signature | 2.8 | glycerol-3-phosphate dehydrogenase |
| 3.95 | Ribosomal protein L2 signature | 2.7 | Hydrophobic region of a signal peptide |
| 3.38 | Region of a membrane-bound protein predicted to be outside the membrane, in the extracellular region | 2 | Mitochondrial carrier protein |
| 2.2 | glycerol-3-phosphate dehydrogenase (NAD(+)) | -6.2 | Region of a membrane-bound protein predicted to be outside the membrane, in the extracellular region |
| -4.9 | Basic-leucine zipper (bZIP) domain signature | -4.4 | Region of a membrane-bound protein predicted to be embedded in the membrane |
| -4.3 | 2,3-bisphosphoglycerate-dependent phosphoglycerate mutase | -4.1 | Arginase family: Agamatinase |
| -3.7 | Carbohydrate binding module (family 6) | -3.8 | Region of a membrane-bound protein predicted to be outside the membrane, in the cytoplasm. |
| -3.24 | Region of a membrane-bound protein predicted to be embedded in the membrane |  |  |
| -3 | Hydrophobic region of a signal peptide |  |  |
| -2.9 | N-terminal region of a signal peptide |  |  |
| -2.8 | UDP-glucose/GDP-mannose dehydrogenase family |  |  |
| -2.5 | Region of a membrane-bound protein predicted to be outside the membrane, in the cytoplasm |  |  |
| -2.5 | Region of a membrane-bound protein predicted to be outside the membrane, in the extracellular region |  |  |
| -2.46 | Region of a membrane-bound protein predicted to be outside the membrane, in the extracellular region |  |  |
| -2.36 | Right handed beta helix region |  |  |
| -2.22 | Region of a membrane-bound protein predicted to be outside the membrane, in the extracellular region |  |  |
| -2 | PhoD-like phosphatase |  |  |

Table S3**:** Most up/down- regulated DEGs identified by DESeq2 with annotation table based on highest and lowest LFC-value comparing SN at 6°C 19h light and 4°C 24h light. The second and fourth column is the gene product described from the output annotation file from the PFAM database. All genes have a padj value of <0.05.

| **Genotype: South-Norway** | | | |
| --- | --- | --- | --- |
| **Condition 6°C 17h light** |  | **Condition**  **4°C 24h light** |  |
| **LFC-value** | **Gene product** | **LFC-value** | **Gene product** |
| 5.45 | EF-hand calcium-binding domain | 22.7 | Reverse transcriptase (RNA-dependent DNA polymerase) |
| 4.07 | Region of a membrane-bound protein predicted to be outside the membrane, in the extracellular region | 7.2 | Zn(2)-Cys(6) binuclear cluster domain |
| 3.3 | Region of a membrane-bound protein predicted to be outside the membrane, in the cytoplasm | 6.7 | Region of a membrane-bound protein predicted to be embedded in the membrane |
| 2.4 | C-terminal region of a signal peptide | 6.2 | Region of a membrane-bound protein predicted to be outside the membrane |
| 2.4 | Hydrophobic region of a signal peptide | 6.12 | Region of a membrane-bound protein predicted to be outside the membrane |
| 2.4 | Region of a membrane-bound protein predicted to be outside the membrane | 5.6 | Signal peptide region |
| -8.52 | Region of a membrane-bound protein predicted to be embedded in the membrane | 5.1 | Region of a membrane-bound protein predicted to be embedded in the membrane |
| -4.8 | Hydrophobic region of a signal peptide | 5.1 | Region of a membrane-bound protein predicted to be embedded in the membrane |
| -4.1 | Region of a membrane-bound protein predicted to be embedded in the membrane | 4.8 | Right handed beta helix region |
| -3.5 | Right handed beta helix region | 4.4 | Region of a membrane-bound protein predicted to be embedded in the membrane |
| -3.42 | Heat shock hsp70 proteins family signature | -14.5 | Glucose / Sorbosone dehydrogenase |
| -2.67 | Pantoate-beta-alanine | -13 | N-terminal region of a signal peptide |
| -2.32 | Region of a membrane-bound protein predicted to be embedded in the membrane | -7 | Glutathione S-transferase, C-terminal |
| -2.25 | Region of a membrane-bound protein predicted to be embedded in the membrane | -5.9 | Actin signature |
| -2.42 | GDP-mannose 4,6 dehydratase | -4.65 | ADP-ribosylation factor family |
| **-**2.07 | Region of a membrane-bound protein predicted to be outside the membrane, in the extracellular region | -4.2 | Glycosyl transferases group |
|  |  | -4.1 | Hydrophobic region of a signal peptide |
|  |  | -3.83 | Right handed beta helix region |
|  |  | -2.8 | Heat shock hsp70 proteins family signature |

Table S4: Table showing the distribution of the top 10 GO terms related to biological processes, cellular component and molecular function for NN at 9°C/17h light. Each term is ordered by the number of significant genes that were found in relation to the expected number of genes associated with the GO term. The expected value is calculated by a hypergeometric distribution statistical model. The column with classicFisher refers to the p-value from the Fisher’s exact test, with a low value indicating that the enrichment of a given GO term is not likely to have happened by chance.

| ***Biological Process*** | ***GO ID*** | ***Term*** | *Annotated* | *Significant* | *Expected* | *classicFisher* |
| --- | --- | --- | --- | --- | --- | --- |
| 1 | GO:0006518 | peptide metabolic process | 185 | 43 | 13.8 | 8.9e-14 |
| 2 | GO:0006412 | translation | 168 | 39 | 12.54 | 2.3e-12 |
| 3 | GO:0043603 | cellular amide metabolic process | 219 | 45 | 16.34 | 2.4e-12 |
| 4 | GO:0043043 | peptide biosynthetic process | 170 | 39 | 12.68 | 3.5e-12 |
| 5 | GO:0043604 | amide biosynthetic process | 191 | 41 | 14.25 | 8.3e-12 |
| 6 | GO:1901566 | organonitrogen compound biosynthetic process | 328 | 52 | 24.47 | 1.0e-09 |
| 7 | GO:0002181 | cytoplasmic translation | 77 | 19 | 5.75 | 1.1e-06 |
| 8 | GO:0044271 | cellular nitrogen compound biosynthetic process | 507 | 61 | 37.83 | 1.6e-06 |
| 9 | GO:1901564 | organonitrogen compound metabolic process | 710 | 71 | 52.98 | 0.00020 |
| 10 | GO:0010467 | gene expression | 554 | 59 | 41.34 | 0.00024 |
| ***Cellular component*** | ***GO ID*** | ***Term*** | *Annotated* | *Significant* | *Expected* | *classicFisher* |
| 1 | GO:0005840 | ribosome | 95 | 33 | 7.64 | 7.0e-15 |
| 2 | GO:0009507 | chloroplast | 195 | 47 | 15.68 | 2.9e-14 |
| 3 | GO:0009536 | plastid | 199 | 47 | 16 | 6.8e-14 |
| 4 | GO:0044391 | ribosomal subunit | 81 | 29 | 6.51 | 1.8e-13 |
| 5 | GO:0009526 | plastid envelope | 80 | 26 | 6.43 | 5.3e-11 |
| 6 | GO:0009532 | plastid stroma | 75 | 25 | 6.03 | 7.1e-11 |
| 7 | GO:0009570 | chloroplast stroma | 75 | 25 | 6.03 | 7.1e-11 |
| 8 | GO:0009579 | thylakoid | 57 | 21 | 4.58 | 3.5e-10 |
| 9 | GO:0009534 | chloroplast thylakoid | 47 | 19 | 3.78 | 4.1e-10 |
| 10 | GO:0031976 | plastid thylakoid | 47 | 19 | 3.78 | 4.1e-10 |
| ***Molecular Function*** | ***GO ID*** | ***Term*** | *Annotated* | *Significant* | *Expected* | *classicFisher* |
| 1 | GO:0003735 | structural constituent of ribosome | 81 | 31 | 6.3 | 4.0e-16 |
| 2 | GO:0005198 | structural molecule activity | 112 | 32 | 8.71 | 1.9e-12 |
| 3 | GO:0015078 | proton transmembrane transporter activity | 11 | 6 | 0.85 | 6.4e-05 |
| 4 | GO:0003723 | RNA binding | 229 | 33 | 17.8 | 8.8e-05 |
| 5 | GO:0015252 | proton channel activity | 4 | 3 | 0.31 | 0.0017 |
| 6 | GO:0046933 | proton-transporting ATP synthase activity | 4 | 3 | 0.31 | 0.0017 |
| 7 | GO:0044769 | ATPase activity, coupled to transmembrane movement of ions, rotational mechanism | 5 | 3 | 0.39 | 0.0041 |
| 8 | GO:0019843 | rRNA binding | 28 | 7 | 2.18 | 0.0042 |
| 9 | GO:0016874 | ligase activity | 37 | 8 | 2.88 | 0.0058 |
| 10 | GO:0008121 | ubiquinol-cytochrome-c reductase activity | 2 | 2 | 0.16 | 0.0060 |

Table S5: Table showing the distribution of the top 10 GO terms related to biological processes, cellular component and molecular function for NN at 6°C/19h light. Each term is ordered by the number of significant genes that were found in relation to the expected number of genes associated with the GO term.

| ***Biological Process*** | ***GO.ID*** | ***Term*** | *Annotated* | *Significant* | *Expected* | *classicFisher* |
| --- | --- | --- | --- | --- | --- | --- |
| 1 | GO:0006518 | peptide metabolic process | 185 | 39 | 12.64 | 2.4e-12 |
| 2 | GO:0006412 | Translation | 168 | 36 | 11.48 | 1.5e-11 |
| 3 | GO:0043043 | peptide biosynthetic process | 170 | 36 | 11.62 | 2.2e-11 |
| 4 | GO:0043603 | cellular amide metabolic process | 219 | 41 | 14.97 | 3.4e-11 |
| 5 | GO:0043604 | amide biosynthetic process | 191 | 38 | 13.05 | 3.7e-11 |
| 6 | GO:1901566 | organonitrogen compound biosynthetic process | 328 | 49 | 22.42 | 9.5e-10 |
| 7 | GO:0044271 | cellular nitrogen compound biosynthetic process | 507 | 59 | 34.65 | 1.8e-07 |
| 8 | GO:0002181 | cytoplasmic translation | 77 | 18 | 5.26 | 1.4e-06 |
| 9 | GO:1901564 | organonitrogen compound metabolic process | 710 | 67 | 48.52 | 7.3e-05 |
| 10 | GO:0034645 | cellular macromolecule biosynthetic process | 472 | 49 | 32.26 | 0.00021 |
| ***Cellular Component*** | ***GO.ID*** | ***Term*** | *Annotated* | *Significant* | *Expected* | *classicFisher* |
| 1 | GO:0005840 | ribosome | 95 | 31 | 7.12 | 4.9e-14 |
| 2 | GO:0009507 | chloroplast | 195 | 44 | 14.62 | 2.0e-13 |
| 3 | GO:0044391 | ribosomal subunit | 81 | 28 | 6.08 | 2.1e-13 |
| 4 | GO:0009536 | plastid | 199 | 44 | 14.92 | 4.4e-13 |
| 5 | GO:0009532 | plastid stroma | 75 | 23 | 5.62 | 7.0e-10 |
| 6 | GO:0009570 | chloroplast stroma | 75 | 23 | 5.62 | 7.0e-10 |
| 7 | GO:0015934 | large ribosomal subunit | 49 | 18 | 3.67 | 2.4e-09 |
| 8 | GO:1990904 | ribonucleoprotein complex | 237 | 42 | 17.77 | 4.8e-09 |
| 9 | GO:0022626 | cytosolic ribosome | 71 | 21 | 5.32 | 9.2e-09 |
| 10 | GO:0009526 | plastid envelope | 80 | 22 | 6 | 1.8e-08 |
| ***Molecular Function*** | ***GO.ID*** | ***Term*** | *Annotated* | *Significant* | *Expected* | *classicFisher* |
| 1 | GO:0003735 | structural constituent of ribosome | 81 | 30 | 5.97 | 7.4e-16 |
| 2 | GO:0005198 | structural molecule activity | 112 | 31 | 8.26 | 2.4e-12 |
| 3 | GO:0015078 | proton transmembrane transporter activity | 11 | 6 | 0.81 | 4.7e-05 |
| 4 | GO:0003723 | RNA binding | 229 | 31 | 16.88 | 0.00018 |
| 5 | GO:0015252 | proton channel activity | 4 | 3 | 0.29 | 0.00147 |
| 6 | GO:0046933 | proton-transporting ATP synthase activity | 4 | 3 | 0.29 | 0.00147 |
| 7 | GO:0019843 | rRNA binding | 28 | 7 | 2.06 | 0.00310 |
| 8 | GO:0044769 | ATPase activity, coupled to transmembrane movement of ions, rotational mechanism | 5 | 3 | 0.37 | 0.00348 |
| 9 | GO:0008121 | ubiquinol-cytochrome-c reductase activity | 2 | 2 | 0.15 | 0.00538 |
| 10 | GO:0016679 | oxidoreductase activity, acting on diphenols and related substances as donors | 2 | 2 | 0.15 | 0.00538 |

Table S6: DEGs related to porphyrin and chlorophyll metabolism. NN = North Norway, MN = Mid Norway, SN = South Norway reference replicates under different temperature and photoperiod conditions. Listed genes with numbers in bold are differentially expressed LFC>2. All genes have a padj-value of <0.05.

| ***Contig name*** | ***Gene product*** | *NN*  *9C17H* | *NN*  *6C19H* | *MN*  *9C17H* | *MN*  *4C24H* | *SN*  6C19H | *SN*  *4C24H* |
| --- | --- | --- | --- | --- | --- | --- | --- |
| **Porphyrin and chlorophyll metabolism** |  |  |  |  |  |  |  |
| contig1009.88.1 | Pheophorbide a oxygenase | -0.70 | -1.40 | -1.20 | 0.04 | -0.29 | 0.04 |
| contig326.9860.1 | Geranylgeranyl reductase | 0.90 | 0.50 | 0.65 | 0.02 | -0.12 | -0.32 |
| contig2822.8817.1  contig89.16737.1 | Magnesium chelatase ATPase  Coproporphyrinogen III oxidase | **4.43**  1.54 | **6.20**  1.21 | **3.76**  NS | 0.76  NS | 0.25  NS | -1.14  NS |

Table S7: DEGs related to ribosomal proteins. NN = North Norway, MN = Mid Norway, SN = South Norway reference replicates under different temperature and photoperiod conditions. Listed genes with numbers in bold are differentially expressed LFC>2. All genes have a padj-value of <0.05. If we like it better as table and not as a supp table we remove it from here (see table 3).

| ***Contig*** | ***Gene Product*** | *NN*  *9C17H* | *NN*  *6C19H* | *MN*  *4C24H* | *MN*  *9C17H* | *SN*  *4C24H* | *SN*  *6C19H* |
| --- | --- | --- | --- | --- | --- | --- | --- |
| contig2822.8809.1 | Ribosomal protein S2 signature | **8.29** | **6.36** | NS | **5.40** | NS | NS |
| contig2461.7725.1 | Ribosomal protein L1p/L10e | **8.05** | **6.84** | NS | NS | NS | NS |
| contig2822.8818.1 | 30S ribosomal protein S16 | **7.85** | **6.28** | NS | NS | NS | NS |
| contig2461.7723.1 | Ribosomal protein L9 | **7.27** | **6.68** | NS | NS | NS | NS |
| contig2822.8819.1 | 30S ribosomal protein S4 | **7.06** | **7.36** | NS | NS | NS | NS |
| contig1308.2337.1 | Ribosomal protein S7 signature | **5.95** | NS | NS | NS | NS | NS |
| contig238.7448.1 | 30S ribosomal protein S14 type Z | **5.64** | **5.52** | NS | NS | NS | NS |
| contig189.5420.1 | Ribosomal L39 protein | **5.62** | **5.41** | NS | NS | NS | NS |
| contig2461.7736.1 | Ribosomal protein L2 signature | **5.49** | **4.47** | NS | **3.95** | NS | NS |
| contig4328.11696.1 | Ribosomal protein L21 signature | **5.48** | **6.18** | NS | NS | NS | NS |
| contig2461.7735.1 | Ribosomal protein L16 signature | **5.19** | **4.45** | NS | NS | NS | NS |
| contig2461.7728.1 | 30S ribosomal protein S12 | **5.06** | **4.65** | NS | NS | NS | NS |
| contig3391.10135.1 | 30S ribosomal protein S7 | **4.97** | **4.32** | NS | NS | NS | NS |

Table S8: DEGs related to proteases. NN = North Norway, MN = Mid Norway, SN = South Norway reference replicates under different temperature and photoperiod conditions. Listed genes with numbers in bold are differentially expressed LFC>2. All genes have a padj-value of <0.05.

| ***Contig name*** | ***Gene Product*** | *NN*  *9C17H* | *NN*  *6C19H* | *MN*  *4C24H* | *MN*  *9C17H* | *SN*  *4C24H* | *SN.*  *6C19H* |
| --- | --- | --- | --- | --- | --- | --- | --- |
| contig1340.2532.1 | CPBP intramembrane metalloprotease | **2.80** | **2.60** | NS | NS | NS | NS |
| contig26.8124.1 | Clp protease catalytic subunit P | 1.90 | 1.30 | NS | NS | NS | NS |
| contig370.10639.1 | ATP-dependent Clp protease adaptor protein | **2.60** | **2.30** | NS | NS | NS | NS |
| contig3385.10113.1 | ATP-dependent Clp protease ATP-binding subunit | **7.40** | **7.10** | NS | NS | NS | NS |
| contig209.6322.1 | ATP-dependent protease HslVU, peptidase subunit | **2.50** | **2.20** | NS | NS | NS | NS |
| contig606.13893.1 | OTU-like cysteine protease | **2.20** | **2.00** | NS | NS | NS | NS |
| contig426.11559.1 | Ubiquitin protease | **2.30** | **2.40** | NS | NS | NS | NS |

| Condition | | **North-Norway Genotype** | **Mid-Norway Genotype** | **South-Norway Genotype** |
| --- | --- | --- | --- | --- |
|  |  | **9°C 17h light 6°C 19h light** | **4°C 24h light 9°C 17h light** | **4°C 24h light 6°C 19h light** |
| Metabolic process | ↑  ↓ | 22 20 13 15 29  8 30 | | |
| Biosynthetic process | ↑  ↓ | 13 13 4 6  6 14 | | |
| Protein metabolic process | ↑  ↓ | 5 4 2 | | |
| Photosynthesis | ↑  ↓ | 1 | | |
| Ribosome | ↑  ↓ | 9 9 10 | | |
| Translation | ↑  ↓ | 3 3 2 | | |
| Transporter activity | ↑  ↓ | 11 2 | | |
| Catalytic activity | ↑  ↓ | 12 19 12 24  8 9 | | |
| Transferase activity | ↑  ↓ | 2 6 3 19  8 6 | | |
| Transport | ↑  ↓ | 3 5 | | |
| DNA metabolic process | ↑  ↓ | 2 1 | | |
| RNA binding | ↑  ↓ | 4 3  1 | | |
|  |  |  | | |

Table S9: Classification of up/down- regulated functional groups of significantly enriched GO-terms for NN, MN and SN from cateGOrizer
